# Clinical Presentation of COVID-19: Case Series and Review of the Literature

**DOI:** 10.3390/ijerph17145062

**Published:** 2020-07-14

**Authors:** Margherita Macera, Giulia De Angelis, Caterina Sagnelli, Nicola Coppola, Vanvitelli COVID-19 Group

**Affiliations:** Department of Mental health and Public Medicine, University of Campania, 80131 Naples, Italy; macera.margherita@libero.it (M.M.); giulia.e19@gmail.com (G.D.A.); caterina.sagnelli@unicampania.it (C.S.)

**Keywords:** COVID-19, SARS-CoV-2, clinical presentation, natural history

## Abstract

COVID-19 infection has a broad spectrum of severity ranging from an asymptomatic form to a severe acute respiratory syndrome that requires mechanical ventilation. Starting with the description of our case series, we evaluated the clinical presentation and evolution of COVID-19. This article is addressed particularly to physicians caring for patients with COVID-19 in their clinical practice. The intent is to identify the subjects in whom the infection is most likely to evolve and the best methods of management in the early phase of infection to determine which patients should be hospitalized and which could be monitored at home. Asymptomatic patients should be followed to evaluate the appearance of symptoms. Patients with mild symptoms lasting more than a week, and without evidence of pneumonia, can be managed at home. Patients with evidence of pulmonary involvement, especially in patients over 60 years of age, and/or with a comorbidity, and/or with the presence of severe extrapulmonary manifestations, should be admitted to a hospital for careful clinical-laboratory monitoring.

## 1. Introduction

Coronaviruses are enveloped viruses with a positive-sense single-stranded RNA genome belonging to the Coronaviridae family, the Nidovirales order, and broadly distributed in humans and other mammals [1]. Although most human coronavirus infections are mild, the epidemics of the two beta-coronaviruses, severe acute respiratory syndrome coronavirus (SARS-CoV) and Middle East respiratory syndrome coronavirus (MERS-CoV) [2], caused more than 10,000 cumulative cases in the past two decades, respectively in 2002 and 2012, with mortality rates of 10% for SARS-CoV and 37% for MERS-CoV [3].

Since December 2019, a new zoonotic beta-corona virus (SARS-CoV-2) has spread all over the world from Wuhan, China [4], causing a disease known as coronavirus disease (COVID-19). On 30 January 2020, the World Health Organization (WHO) declared a public health emergency [5], and the epidemic rapidly evolved into a pandemic by March 2020 [6], with a high number of cases in the European Region, especially in Italy [7]. 

SARS-CoV-2 is able to enter host cells through the binding between the viral structural spike (S) protein and the angiotensin-converting enzyme 2 (ACE2) receptor, present in the lung and in other tissues [8]. Viral entry is facilitated by a type 2 transmembrane serine protease, TMPRSS2, via the S protein [9]. Once binding between the S protein and receptor is established, the virus particles enter the host cell through membrane fusion and endocytosis. Inside the cell, the viral genome is released and translated into viral polypeptides, which are then cleaved into small products by proteases. The following stages include RNA synthesis by RNA-dependent RNA polymerase (RdRp), structural protein synthesis, exocytosis, and the release of the new assembled virions [8]. COVID-19 infection has a broad spectrum of severity ranging from an asymptomatic form to a severe acute respiratory syndrome that requires mechanical ventilation. The early presentation of COVID-19 infection is typically non-specific. Among symptomatic patients, about 80% showed a mild clinical course [10] characterized by a dry cough, sore throat, low-grade fever, or malaise; in 20% of cases, the general condition worsened in about seven days from the beginning of the symptoms, culminating in respiratory failure [11,12].

Given the wide spectrum of clinical presentation, demographic, clinical, and biochemical criteria are needed to identify the individuals most likely to progress to a severe illness. Starting from the description of our case series, we evaluated the clinical presentation and evolution of COVID-19. This article is addressed particularly to physicians caring for patients with COVID-19 in their clinical practice. The intent is to identify the subjects in whom the infection is most likely to evolve and the best methods of management in the early phase of infection to determine which patients should be hospitalized and which could be monitored at home.

## 2. Methods

We conducted comprehensive computerized literature research to identify studies analyzing diagnostic tests for COVID-19 using MEDLINE and EMBASE from January 2020 to 15 May 2020, involving both medical subject heading (MeSH) terminology and relevant keywords for search strings. The following items were used to search for the studies: “clinical characteristics,” “natural history,” “COVID-19,” and “SARS-CoV-2.” We performed this research to further the knowledge of the clinical presentation and natural history of COVID-19.

## 3. Statistical Analysis

In case series analysis, continuous variables were expressed as median (IQR) and compared with the Mann–Whitney U test; categorical variables were expressed as a number (%) and compared by χ^2^ test or Fisher’s exact test. A *p*-value of <0.05 was considered to be statistically significant.

## 4. Case Series

We described the first 40 subjects with SARS-CoV-2 rt-PCR positive based on nasopharyngeal swabs observed from 8 March 2020 to 31 March 2020 at the Vanvitelli Covid Unit in Naples, southern Italy. Table 1 shows the demographic and clinical characteristics of the patients enrolled. The median age of patients was 52 years (IQR, 41.25–65.75), and 20 were males. Of the 40 patients, 22 (55%) had one or more coexisting medical conditions: hypertension in 17 (42%), cardiovascular disease in 8 (20%), diabetes mellitus in 4 (10%), malignancy in 4 (10%), and chronic respiratory disease in 4 (10%).

Of the 40 patients enrolled, 3 (7.5%) were asymptomatic, and 37 patients (92.5%) were symptomatic. All the symptoms were reported by the patients and confirmed by the physicians. Among the symptomatic patients, the most common symptoms at the onset of illness were fever (in 31 (77%)), defined as an axillary temperature of 37.5 °C or higher, fatigue (in 24 (60%)), myalgia (in 23 (58%)), lack of appetite (in 23 (58%)), and dry cough (in 15 (37%)). Other symptoms were diarrhea (in eight (20%)), anosmia (in 12 (30%)), dysgeusia/ageusia (in 13 (33%)), nausea (in three (8%)), rhinorrhea (in 2 (5%)), conjunctivitis (in 2 (5%)), and skin lesions (in 2 (5%)).

Of the 40 patients enrolled, 24 (60%) were in home isolation and 16 (40%) hospitalized. The decision of home isolation was made by the physician. The median duration from the first symptoms to hospital admission was 8.5 days (IQR 6.5–11). Table 1 shows the demographic characteristics of the two groups of patients. Compared with the 24 patients in home isolation, the 16 hospitalized patients were significantly older (median age, 69 years (IQR, 48.5–80.25) vs. 43.5 years (IQR, 39.75–55.25); *p* = 0.001) and had more probable underlying comorbidities (75% vs. 42%; *p* = 0.05). Hypertension and malignancy were more frequently detected in hospitalized patients (75% vs. 21%, *p* = 0.001; 25% vs. 0, *p* = 0.02, respectively).

Of the 24 patients in home isolation, 21 (88%) were symptomatic, while all hospitalized patients were symptomatic. In the 24 patients in home isolation, the most frequent symptoms were fever (in 17 (71%)), asthenia (in 15 (63%)), loss of appetite (in 15 (63%)), myalgia (in 15 (63%)), ageusia/dysgeusia (in 8 (33%)), and cough (in 6 (25%)). In the 16 hospitalized patients, the same symptoms were observed, but cough (in 9 (56%)), dyspnea (in 5 (33%)), and diarrhea (in 6 (38%)) were more frequently observed as clinical manifestations of SARS-CoV-2 infection. Clinical or imaging signs of pulmonary involvement were observed in 14 (88%) hospitalized patients and in none in home isolation. 

To date, all patients in home isolation recovered within day 30 from the onset of symptoms, and 20 patients cleared the virus, as demonstrated by the rt-PCR negativity for SARS-CoV-2 in two nasopharyngeal swabs; among the hospitalized patients, 14 recovered and cleared the virus, while two patients died. The median time that elapsed from the first positive swab to a negative swab was 22 days (IQR, 12.25–32) for patients in home isolation and 22.5 days (IQR, 17.5–32.75) for hospitalized patients.

## 5. Review of Literature

### 5.1. Clinical Presentation of COVID-19

#### 5.1.1. Typical Clinical Manifestations

The incubation period for SARS-CoV-2 was estimated as 2–14 days, according to publicly available data; 14 days has been chosen as the cut-off for self-quarantine [13,14]. Guan et al. demonstrated that the median incubation period was four days and that 95% of the 1099 hospitalized patients enrolled (median age was 47 years; 41.9% were female) developed the symptoms within 10 days [15]. 

Another study of 72,314 Chinese patients, conducted by the Chinese Center for Disease Control and Prevention, reported that 1% were asymptomatic cases [16], while a study with a mathematical model estimated that the percentage of subjects infected but not confirmed was 86% (95% CI: 81.5–89.8%) [17]. The transmission of COVID-19 through patients who have not yet developed symptoms was observed in many reports, although the symptoms were absent [18,19,20]. In the symptomatic subjects, early-phase fever was present in 45%, and constitutional symptoms, such as muscle or bone aches, chills, headache, sore throat, and nasal congestion, were observed [21]. The symptomatic patients may have shown a mild clinical evolution or the development of pulmonary involvement [22].

In the first group of patients with mild symptoms, nasal congestion and sputum were the most common (34.3% and 39.5% respectively), while fever was observed only in 11.6% [23]. Radiological abnormalities on computer tomography (CT) were usually not observed [21,22,24,25,26]. However, some patients who had initially mild symptoms subsequently showed a precipitous clinical deterioration that occurred approximately one week after onset of symptoms [26,27].

When there was lung involvement, respiratory symptoms, such as dyspnea or cough and sputum, were present [21]. In these patients, CT showed a range of features including ground-glass opacities, interstitial infiltration, crazy-paving pattern, and multiple patchy consolidations in both lung fields; in addition, vessel enlargement, thick interlobar septa, and air bronchograms were observed [22]. Clinically, in severe pneumonia, a respiratory rate of at least 30/min, SpO_2_ 93%, or PaO_2_/FiO_2_ 300 mmHg was observed [28].

As regards the biochemical data in COVID-19 patients, leuco-lymphopenia, thrombocytopenia, hypoalbuminemia, and elevated lactate dehydrogenase were observed. Most of the patients also had elevated levels of C-reactive protein; less common were elevated levels of alanine aminotransferase, aspartate aminotransferase, creatine kinase, and D-dimer [25,29,30].

#### 5.1.2. Atypical Clinical Manifestations

The ability of the virus to bind the ubiquitous ACE2 receptors allows SARS-CoV-2 to target organs other than the lungs. ACE2 is highly expressed in absorptive intestinal epithelial cells, in the ileum and colon, as well as in cholangiocytes, hepatocytes, and esophageal cells. This explains the presence of gastrointestinal symptoms, such as diarrhea, nausea, and vomiting, and elevated liver function test results. Considering the 1602 patients enrolled in 10 different case series, 55 had diarrhea (average 5.6%, range 2–33.98%), and 72 had nausea or vomiting symptoms (average 4.49%, range 1–10%). All of these patients were predominantly male and were hospitalized [21,26,31]. A recent study found that almost half of the 99 hospitalized patients infected with COVID-19 showed liver involvement; the cause of elevated aminotransferase serum levels remains unclear, but it may be due to liver damage by COVID-19 or by antiviral drugs [25] (Table 2). In our case series, eight (20%) patients had diarrhea, but only one (3%) patient had increased aminotransferase serum levels.

The cardiovascular system may also be involved in COVID-19, as ACE-2 receptors play an important role in its neuro-humoral regulation. In fact, acute cardiac injury, as demonstrated by a significant elevation of cardiac troponins, occurred in approximately 8–12% of COVID-19 patients [32], probably due to virus-related damage and/or the effect of systemic inflammation [33]. Another life-threatening cardiac involvement is fulminant myocarditis, as suggested by case reports [34,35,36]. Moreover, in a Chinese study on 138 COVID-19 patients, a prevalence of arrhythmia in 16.7% was reported [37] (Table 2). In our case series, an increase in cardiac troponins was observed in four (10%) patients, arrhythmias in four (10%) patients, while no patient experienced fulminant myocarditis.

Elevated D-dimer levels, which may suggest pulmonary embolism, were observed in 36–46.4% of patients with COVID-19 [38]. However, we noted that pulmonary embolism should be confirmed by a pulmonary angio-CT. A viral infection with subsequent systemic inflammatory response probably leads to an imbalance between pro-coagulative and anti-coagulant mechanisms [39] (Table 2). In our case series, elevated D-dimer levels were observed in 7 (17%) patients.

Recently, dermatological manifestations were also observed in COVID-19 patients. In a study by Recalcati et al., 20.4% of the 88 COVID-19 patients developed cutaneous manifestations during the disease [40]; it was found that most cutaneous presentations were erythematous rash (77.8%) with a few cases of urticaria (16.7%) and vesicle formation (5.6%). Although the pathogenetic mechanisms are still unclear, they may be due to a secondary consequence of infection or a primary infection of the skin itself (Table 2). In our case series, only two (5%) patients had cutaneous manifestations: specifically, maculo-papular exanthema in both patients. In one patient, this extended to the trunk, root of the limbs, and scalp.

The evidence of central nervous system (CNS) involvement of COVID-19 is scanty. However, some reports suggest that SARS-CoV-2 may present neurological manifestations, such as the loss of smell and taste, ataxia, confusion, and headache [41,42,43]. A few patients showed seizure or cerebrovascular disease [44]. The hematogenous route appears to be the most likely pathway for SARS-CoV2 to reach the brain, but other routes, such as across the cribriform plate of the ethmoid bone in proximity to the olfactory bulb, should be taken into consideration in patients who exhibit loss of smell and taste [45,46]. In our case series, 12 (30%) patients reported hyposmia, while 13 (32.5%) reported ageusia. None of the patients complained of confusion, headache, ataxia, or convulsions (Table 2).

### 5.2. Correlation between Clinical Presentation and Clinical Evolution

According to WHO reports, the overall fatality rate for COVID-19 is estimated at 2.3% [47], but the fatality rate has varied among studies from 1.4% to 4.3% [21,37]. In our case series, the overall mortality rate was 2.5%. The differences in the results among different studies may be due to the study population (symptomatic and asymptomatic, hospitalized or home isolation) as well as the differences among the studies in terms of underlying chronic diseases and median age of subjects enrolled.

Although the risk factors of COVID-19 remain unclear, many studies reported that a significant proportion of patients had underlying conditions [21,37]. Chen et al. showed that 50.5% of 51 COVID-19 patients had a chronic disease, namely cardiovascular and cerebrovascular (40.4%) [25]. Of 1099 patients with SARS-CoV-2 infection, Guan et al. showed that 23.2% had at least one underlying disease; hypertension was the most common (14.9%), followed by diabetes mellitus (7.4%) [15]. Another large study of COVID-19 cases of varying degrees of severity showed that hypertension was the most common underlying disease (2608, 12.8%), followed by diabetes mellitus (1102 patients, 5.3%) and cardiovascular disease (873 patients, 4.2%). All patients were predominantly male [47] (Table 3).

Moreover, patients with severe COVID-19 were more likely to have comorbidities than patients with non-severe diseases (37.6% vs. 20.5%) [21]. A similar trend was observed in another study of 138 hospitalized patients with SARS-CoV-2 pneumonia, in which 46.4% had comorbidities, and intensive care unit (ICU) patients were more likely to have underlying diseases compared to non-ICU patients (72.2% vs. 37.3%, *p* < 0.001) [37] (Table 3).

Other factors associated with an elevated case fatality rate included male sex, higher age, baseline diagnosis of severe pneumonia, and delay in diagnosis [47]. The China CDC reported that patients aged over 80 years had the highest case fatality rate (14.8%) [47]. As regards the biochemical data associated with severe forms, the data are not conclusive. A procalcitonin value of more than 0.5 ng/mL was associated with a higher risk of progression to a critical illness, such as an increase during the disease in total white blood cells compared to the baseline value [48,49]. In our case series, the hospitalized patients were significantly older and more likely to have underlying comorbidities, especially hypertension and malignancy, than those in home isolation.

## 6. Conclusions

**Figure 1:**
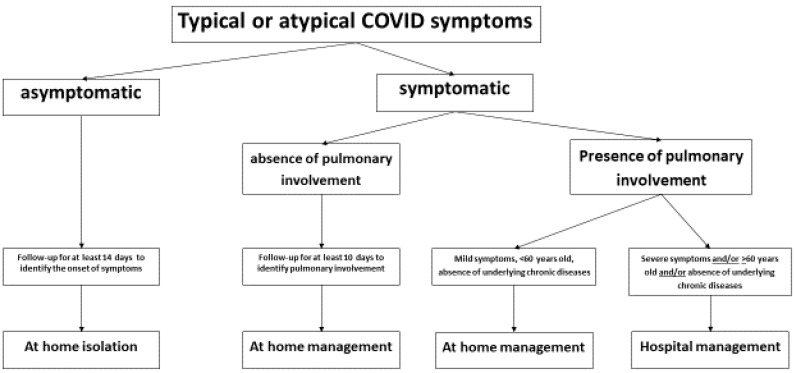
Management of COVID-19 patients according to the clinical presentation.

COVID-19 may present a varied clinical picture, such as asymptomatic carriage, with or without associated pneumonia, and with or without several extrapulmonary manifestations [50,51,52]. Figure 1 shows a possible management plan for patients according to their clinical presentation. Asymptomatic patients with nasopharyngeal swabs positive for SARS-CoV-2 rt-PCR should be followed for 14 days to evaluate the appearance of symptoms. Similarly, patients with mild symptoms arising after more than 10 days, and without evidence of pneumonia, can be managed at home with periodic telephone evaluation. However, patients with evidence of pulmonary involvement, especially in patients over 60 years of age, and/or with a comorbidity, and/or the presence of severe extrapulmonary manifestations, should be admitted to a hospital for careful clinical-laboratory monitoring with periodic blood gas analysis, blood count, liver and kidney function evaluation, dosage of procalcitonin, reactive protein C, and D-dimer. In these patients, it is also important to do a radiological follow-up with lung CT [53].

In conclusion, other studies on the natural history of COVID-19 are needed to identify the correct management of COVID-19 patients and differentiate patients with a favorable or unfavorable clinical course according to the initial clinical presentation.

## Figures and Tables

**Table 1:** Demographic and clinical characteristics of the patients enrolled.

	All	In Home Isolation (a)	Hospitalized (b)	*p*-Value a vs. b
**N° of patients**	40	24	16	
**N° (%) of patients in class:**				
**18–29**	4 (10)	3 (13)	1 (6)	0.63
**30–39**	2 (5)	2 (8)	0	0.50
**40–49**	11 (28)	7 (29)	4 (25)	1.0
**50–59**	8 (20)	6 (25)	2 (13)	0.43
**60–69**	8 (20)	6 (25)	2 (13)	0.43
**>70**	7 (17)	0	7 (44)	0.0006
**Age, years, median (IQR)**	52 (41.25–65.75)	43.5 (39.75–55.25)	69 (48.5–80.25)	0.0017
**N° (%) of males**	20 (50%)	14 (58)	6 (38)	0.33
**N° (%) of patients with comorbidity:**	22 (55)	10 (42)	12 (75)	0.054
**Arterial Hypertension**	17 (42)	5 (21)	12 (75)	**0.0011**
**Diabetes Mellitus**	4 (10)	1 (4)	3 (19)	0.28
**Malignancy**	4 (10)	0	4 (25)	**0.019**
**Chronic Respiratory Disease**	4 (10)	1 (4)	3 (29)	0.28
**Cardiovascular Disease**	8 (20)	4 (17)	4 (25)	0.69
**Renal Insufficiency**	2 (5)	1 (4)	1 (6)	1.0
**Symptoms, N° (%) of subjects:**	37 (92.5)	21 (88)	16 (100)	1.0
**Fever**	31 (77)	17 (71)	14 (88)	0.27
**Cough**	15 (37)	6 (25)	9 (56)	0.093
**Dyspnea**	5 (13)	0	5 (33)	**0.0066**
**Anosmia**	12 (30)	6 (25)	6 (38)	0.48
**Ageusia/Dysgeusia**	13 (33)	8 (33)	5 (32)	1.0
**Diarrhea**	8 (20)	2 (8)	6 (38)	**0.042**
**Nausea**	3 (8)	1 (4)	2 (13)	0.55
**Lack of appetite**	23 (58)	15 (63)	8 (50)	0.52
**Fatigue**	24 (60)	15 (63)	9 (56)	0.75
**Myalgia**	23 (58)	15 (63)	8 (50)	0.52
**Rhinorrea**	2 (5)	1 (4)	1 (6)	1.0
**Conjunctivitis**	2 (5)	0	2 (13)	0.15
**Skin lesions**	2 (5)	1 (4)	1 (6)	1.0
**N° (%) of patients with CT evidence of interstitial pneumonia**	14 (35)	0	14 (88)	<**0.00001**
**Time, days elapsed from the first positive swab to negative swab, median (IQR)**	22.5 (13.75–32)	22.0 (12.2–32.0)	22.5 (17.5–32.7)	0.75

**Table 2:** Studies reporting the atypical clinical presentation of COVID-19.

Author [Ref.]	Country	N° Patients	Males N° (%)	Age, Years Median (Range)	Evidence
**Gastro-intestinal manifestations**					
Guan WJ, et al. [21]	China	1099	640 (58.1%)	47 (35–58)	55 (5.0%) nausea or vomiting 42 (3.8%) diarrhea
Chen N, et al. [25]	China	99	67 (68%)	55 (21–82)	43 (43%)liver function abnormality
Huang C, et al. [26]	China	41	30 (73%)	49 (IQR 41–58)	1 (3%) diarrhea
Pan L, et al. [31]	China	204	107 (52%)	52.9 ± 16	103 (50.5%) digestive symptom 81 (78.6%) lack of appetite35 (34%) diarrhea 4 (3.9%) vomiting 2 (1.9%) abdominal pain
**Cardio-vascular manifestations**					
Chen C, et al. [35]	China	41	30 (73%)	N/A	5 (12%) acute cardiac injury
Wang D, et al. [36]	China	138	75 (54.3%)	56 (22–92)	10 (7.2%) acute cardiac injury
Zhang L, et al. [38]	China	343	169 (49.7%)	68 (18–92)	67 (19%) D-dimer levels over 2.0 µg/mL
Han H, et al. [39]	China	94 cases 40 controls	48 (51%) cases 28 (70%) in the control group	N/A	D-dimer (10.36 vs. 0.26 ng/L; *p* < 0.001), and FDP (33.83 vs. 1.55 mg/L; *p* < 0.001) were higher in case than in control group
**Dermatological manifestations**					
Recalcati S, et al. [40]	Italy	88	N/A	N/A	18 (20%) cutaneous manifestation 14 (77%) erythematous rash 3 (16%) widespread urticaria 1 (5.5%) chickenpox-like vesicles
**Neurological manifestations**					
Mao L, et al. [42]	China	214	87 (40.7%)	52.7 (SD 15.5)	78 (36.4%) neurologic symptoms (more common in patients with severe infection (45.5%) 36 (16.8%) dizziness 28 (13.1%) headache 12 (5.6%) taste impairment11 (5.1%) smell impairment
Helms J, et al. [44]	France	58	N/A	63 (IQR, 37–65)	47(81%) neurologic findings

**Table 3:** Studies evaluating the severe clinical forms of COVID-19.

Author [Ref.]	Country	N° Patients	N° (%) of Males	Age, Years Median (Range)	N° (%) of Severe Forms	N° (%) of Deaths	Factors Associated with Severe Forms
Guan W, et al. [15]	China	1099	640 (58.1%)	47 (35–58) Median (IQR)	173 (15.7%)	15 (1.4%)	Age, presence of any coexisting illness, laboratory abnormalities
Wang D, et al. [37]	China	138	75 (54.3%)	56 (22–92)	36 (26.1%)	6 (4.3%)	Age, comorbidities, pharyngeal pain, dyspnea, dizziness, abdominal pain, anorexia, higher levels of D-dimer, creatine kinase, and creatine
Chen N, et al. [25]	China	99	67 (68%)	55 (21–82)	23 (23%) [ICU]	11 (11%)	Age, smoking, lymphopenia, bilateral pneumonia, hypertension
Huang C, et al. [26]	China	41	30 (73%)	49 (41–58) Median (IQR)	13 (32%) [ICU]	6 (15%)	Higher plasma levels of IL2, IL7, IL10, GSCF, IP10, MCP1, MIP1A, and TNFα, higher prothrombin time and D-dimer level
The Novel Coronavirus Pneumonia Emergency Response Epidemiology Team [47]	China	44,672	22,981 (51.4%)	(30–79)	6168 (13.8%), severe 2087 (4.7%), critical	1023 (2.3%)	Age, male, comorbidities
Fu L, et al. [49]	China	3600 (from 43 studies)	56.50% (from 42 studies)	41 (39–72)	25.6% (from 21 studies)	3.60%	Age, laboratory abnormalities, comorbidities
Liu Z, et al. [22]	China	72	39 (54.2%)	46.2 ± 5.9 (M ± SD)	8 (11.1%)	0	Age, higher lung severity score, lymphopenia
Zhang L, et al. [38]	China	343	169 (49.7%)	68 (18–92)	N/A	13 (3.8%)	Higher D-dimer level
Mao L, et al. [42]	China	214	87 (40.7%)	52.7 ± 15.5 (M ± SD)	88 (41.1%)	N/A	Age, comorbidities (especially hypertension), neurologic manifestations, increased inflammatory response, including higher white blood cell counts, neutrophil counts, lower lymphocyte counts, increased C-reactive protein levels, higher D-dimer level, and multiple organ involvement

## Appendix A

Vanvitelli COVID-19 Group: Nicola Coppola, Caterina Sagnelli, Stefania De Pascalis, Maria Stanzione, Gianfranca Stornaiuolo, Angela Cascone, Salvatore Martini, Margherita Macera, Caterina Monari, Federica Calò, Andrea Bianco, Antonio Russo, Valeria Gentile, Clarissa Camaioni, Giulia De Angelis, Giulia Marino, Roberta Astorri, Ilario De Sio, Marco Niosi, Serena Borrelli, Vincenzo Carfora, Benito Celia, Maria Ceparano, Salvatore Cirillo, Maria De Luca, Marco Di Mauro, Grazia Mazzeo, Marco Migliaccio, Filiberto Fausto Mottola, Giorgio Paoli, Riccardo Ricciolino, Giorgio Spiniello, Nicoletta Verde.
